# Physical activity as clinical practice care for patients with type 2 diabetics and its implementation in routine clinical care: an expert opinion survey

**DOI:** 10.3389/fendo.2025.1518285

**Published:** 2025-09-11

**Authors:** Itai Goldfarb, Ayelet Giladi, Sharon Barak, Ishay Lev, Horesh Dor-Haim

**Affiliations:** ^1^ The Physical Education Department, Kaye Academic College of Education, Be'er-Sheva, Israel; ^2^ The Levinsky-Wingate Academic Center, The Department of Physical Activity for The Elderly, Netanya, Israel; ^3^ Kaplan Medical Center, The Institute for Cardiac Rehabilitation and Prevention of Heart Disease, Rehovot, Israel; ^4^ The Nursing Department, Faculty of Health Sciences, Ariel University, Ariel, Israel; ^5^ The Department of Pediatric Rehabilitation, Chaim Sheba Medical Center at Tel HaShomer, The Edmond and Lily Safra Children’s Hospital, Ramat Gan, Israel; ^6^ Clalit Health Services, Family Medicine Department, Jerusalem, Israel; ^7^ Forest Heart Institute, Malcha Meuhedet Health Services, Jerusalem, Israel

**Keywords:** diabetes, physical activity, exercise training, exercise physiology, family medicine

## Abstract

**Introduction:**

Physical activity (PA) is integral to type 2 diabetes (T2D) treatment, yet few national health services incorporate structured PA services as part of T2D treatment. Moreover, healthcare professionals acknowledge their role in integrating PA into the daily routines, but implementation faces notable barriers. Recognizing the pivotal role of healthcare systems and professionals in promoting physical activity–based interventions is crucial to closing the gap between guidelines and their implementation in real-world settings.

**Methods:**

The study involved 363 healthcare and physical activity professionals across diverse clinical settings. A questionnaire, developed through a focus group and literature review, assessed participants’ attitudes and knowledge on PA and national practices pertaining to PA for people with T2D. The questionnaire’s internal reliability was examined using Cronbach’s alpha. Chi-squared tests compared participants’ attitudes and knowledge prevalence in each healthcare and physical activity professionals’ sector.

**Results:**

Participants (mean age = 48.00 ± 12.55) overwhelmingly supported PA inclusion in healthcare (97.8%) and reimbursement (77%). Translating PA recommendations into clinical practice remains a significant challenge due to several interrelated barriers. These include time constraints that hinder physicians’ ability to provide in-depth counseling during routine visits, and patient-related factors, such as low responsiveness (43.8%) and limited awareness (33.3%). Around 70% of physicians endorsed regular PA recommendations. Exercise physiologists (30%) and physiotherapists (28%) were deemed most qualified to instruct T2D patients, followed by physicians (15.7%). A majority (57%) advocated for a multidisciplinary approach to PA prescription, incorporating aerobic, strength, and stretching training.

**Conclusion:**

The study underscores the need for the National healthcare system to prioritize infrastructure development, including multidisciplinary teams, for personalized PA programs vital to individuals with T2D.

## Introduction

Physical activity (PA) plays a crucial role in preventing and managing type II diabetes (T2D) (1–4). Accordingly, global health organizations advocate diverse strategies to promote PA for individuals with T2D (5–7). Recommendations include raising awareness, implementing educational programs, facilitating personalized PA guidance, and endorsing counseling and training resources within diabetes clinics (4).

PA for T2D patients should incorporate both aerobic and resistance exercises. Each exercise type independently has been proven beneficial for patients with T2D (e.g., contribute to glycemic balance by reducing insulin resistance). However, the most substantial improvement in key glycemic control biomarkers, including a decrease in glycated hemoglobin values, is observed when these two modalities are combined. Therefore, optimal glycemic balance is recommended through the integration of both aerobic and resistance training for comprehensive benefits (8, 9).

Despite the pivotal role of PA in preventing and treating T2D, few national health services within the Organization for Economic Co-operation and Development incorporate structured PA services as part of T2D treatment. Effective health policies that promote PA and lifestyle changes demonstrate significant success in altering unhealthy behaviors (10, 11). Healthcare professionals acknowledge their role in integrating PA into the daily routines of individuals with diabetes, but implementation faces notable barriers (12). Many health professionals do not consistently recommend or assess PA in their clinical care practices (13, 14). Recognizing the crucial role of the healthcare system in advancing PA policies, especially for populations at high risk due to physical inactivity (e.g., individuals with T2D) (15), understanding the attitudes and actions of healthcare providers regarding PA for T2D patients becomes imperative. Therefore, this study aims to examine the attitudes and knowledge of healthcare providers and PA practitioners in various clinical settings, both hospital and community-based, regarding the implementation of PA for patients with T2D.

This study delves into the perspectives of physicians and other professionals regarding the prescription of PA. It aims to understand their views on the framework determining which patients should receive guidance, treatment, and knowledge. The survey incorporates inquiries about key components deemed essential and deserving of reimbursement in PA programs designed for diabetic patients.

## Methods

### Study population

The study encompassed professionals aged 18 and above within the health and exercise/physical activity domain. This included individuals from medical fields (physicians), paramedical roles (e.g., physical therapists, exercise physiologists), and physical activity sectors (e.g., fitness trainers). All participants provided explicit consent for their involvement in the study, and ethical approval was granted by the Helsinki Committee of Chaim Sheba Medical Center, Ramat-gan, Israel (Helsinki registration number: SMC- 7640-20).

### Outcome measures

An anonymous questionnaire was collaboratively developed by a focus group comprising professionals, including physicians, paramedical personnel, and PA experts, along with insights from a literature review. The literature review, sourced from English language articles using keywords like Diabetes, Physical Activity, Exercise Training, Exercise Physiology, and Family Medicine, involved searches on electronic databases such as PubMed, Google Scholar, and Diabetics Officials Central Register.

The questionnaire underwent an iterative development process, including five focus groups conducted via Zoom meetings and conference calls, followed by three email-based rewriting versions. Structured on a rating scale ranging from 1 (strongly disagree) to 5 (strongly agree), the questionnaire addressed several categories: 1) Importance of PA for Patient Health 2) Professional Awareness of PA Guidance for Diabetic Patients 3) Most Qualified Professional for Prescribing PA to Diabetic Patients 4) Suitable Framework for Controlling PA Programs for Diabetic Patients 5) National Infrastructures and Information 6) Key Components to Be Reinforced in Diabetic PA Programs 7) Funding Source for PA Activity for Diabetic Patients 8) Main Factors Hindering Regular PA Among Diabetic Patients.

The final version of the questionnaire (Supplemental file number 1) was established only upon achieving unanimous agreement among the developers. This final version comprises 12 questions designed to assess perceptions and attitudes concerning the role of physical activity in the treatment and prevention of diabetes. The final version of the questionnaire includes a combination of structured response formats. Several items were based on 5-point Likert-type scales to evaluate agreement levels or perceived importance, with anchors ranging from 1 (e.g., “not at all aware,” “not effective at all,” or “strongly disagree”) to 5 (e.g., “very aware,” “very effective,” or “strongly agree”). Other questions used single-choice categorical formats to capture professional opinions, offering mutually exclusive predefined options (e.g., preferred professional to prescribe physical activity, perceived barriers to patient adherence). No open-ended questions were included in the questionnaire.

### Procedures

The survey, presented as a digital link through channels like email, WhatsApp, and text messages, was disseminated between June 22nd, 2020 until July 21st, 2020 to a convenient sample of colleagues and acquaintances within the field. Additionally, national professional bodies, including family physician groups, exercise physiologists, trade unions, and hospital departments, were engaged to facilitate wider distribution of the questionnaire.

To assess test-retest reliability, a subsample of 30 health professionals completed the questionnaire twice, with a one-week interval between administrations. Only the 12 items related to physical activity attitudes and practices were evaluated for reliability; sociodemographic and clinical background questions were not included in this analysis.

### Statistical analysis

Normality testing for continuous variables was conducted. Initially, data distribution was visually assessed using Q-Q plots. This was followed by the Kolmogorov–Smirnov test, where a p-value greater than 0.05 indicated that the data were normally distributed.

### Power analysis

A *post-hoc* power analysis was performed using G*Power (version 3.1) to evaluate the statistical power of the Kruskal–Wallis H test in detecting differences in professional attitudes toward PA. The analysis was based on a total sample size of 363 participants, distributed across seven professional groups, with a significance level (α) of 0.05, and the mean effect size calculated across the five ordinal-scale items.

### Study participants

Demographic and professional characteristics were presented using averages, standard deviations, and participant percentages. For ordinal and categorical variables, median scores and the 25th-75th percentile range were utilized.

### Scale’s psychometric properties

The internal reliability of the survey was assessed using Cronbach’s alpha Index, with an accepted threshold set at more than 0.70 (16). Test-retest reliability of the scale was calculated using the intraclass correlation coefficient (ICC) with a 95% confidence interval. For interpretation of the ICC score, ranging from 0.00 to 1.00, values greater than 0.75 are considered good and values of 0.75 or less are considered poor-to-moderate reliability (17).

The structure of the scale was examined by exploratory factor analysis. The analysis was conducted via the principal component analyses (PCA) extraction method, followed by orthogonal (varimax) rotation to maximize variance. Before conducting the PCA, various statistical assumptions necessary for PCA were tested by the Kaiser-Meyer-Olkin index of sampling adequacy (> 0.75); Bartlett’s test of sphericity (p <.001); multicollinearity (variance inflation factor > 2.5) (18); latent root criteria (eigenvalues > 1.0, Kaiser’s criterion K1); and inspection of the scree plot (19). A factor with three or fewer items was considered unstable. Items with communality values of <.40 were removed and the PCA was repeated.

Cross-loading of items was evaluated. A cross-loading item is defined as an item that loads at.32 or higher on two or more factors (20). Cross-loading items were removed from the analysis and the PCA was repeated. To assess the fit of the factor models, differences between the model-based correlations and observed correlations were examined. No more than 50% of the residuals should be greater than.05.30 Once no communalities, cross-loading, or residual issues were identified, the PCA was complete.

### Response distribution

Response distribution differences were examined using Chi-squared tests, and all statistical analyses deemed significance at p < 0.05 with two-tails. IBM SPSS Statistics software (version 19) facilitated these analyses.

### Differences between health professionals in attitudes towards physical activity in people with diabetes

Differences in attitudes toward physical activity among health professionals were analyzed using the Kruskal-Wallis test for ordinal items, followed by *post-hoc* pairwise comparisons with Bonferroni correction, adjusting the significance threshold to α = 0.01 (0.05 divided by 5 comparisons).

For categorical variables, differences were examined using Chi-square tests, with the Bonferroni correction applied by dividing the standard alpha level by the number of comparisons (α = 0.007, i.e., 0.05/7). To further explore whether healthcare professionals background predicted responses to key items, multiple linear regression analyses were conducted for variables in which significant between-profession differences were observed.

Results are reported using standardized beta coefficients, p-values, and adjusted R² to indicate model fit. Significance was set at p < 0.05.

## Results

The calculated effect sizes ranged from 0.12 for the item “The secondary harm to public health due to sedentary behavior during COVID-19 may be greater than the disease itself” to 0.38 for the item “Physicians routinely recommend daily physical activity to people with diabetes.” The calculated mean effect size used for power analysis was 0.26, representing a small-to-medium effect according to conventional benchmarks.

The resulting statistical power was 0.975, indicating a very high likelihood (97.5%) of detecting between-group differences, if they exist. These results support the adequacy of the sample size for detecting meaningful differences in professional attitudes using non parametric ordinal measures.

Q-Q plot inspection showed substantial deviations from normality, and the Kolmogorov–Smirnov test yielded a significant result (p < 0.05). Therefore, non-parametric statistical analyses were deemed appropriate and were subsequently applied.

### Study participants

The survey garnered responses from 363 healthcare professionals (mean age: 48.00 **±** 12.55; 70.80% women). Participants predominant workplace settings were health maintenance organizations (HMOs) (31.40%), independent practices (22.03%), and public hospitals (20.38%). The survey sample included a diverse range of professionals, with the highest representation from physicians (20.66%) and physiotherapists (17.07%; **Table 1**).

**Table 1:** General characteristics and professional background of the study population (n = 363).

Variable	Mean or Number of patients	SD (rage) or percentage
Age (years): mean	48	12.6 (22.0-82.0)
Gender (n, %)		
Male	257	70.8
Female	106	29.2
Professional seniority (Years)	19.6	12 (0.40-53.0)
^†^Workplace (n, %)		
Health maintenance organization	114	31.4
Independent physician	80	22.03
Hospital public Center	74	20.38
Hospital Private Clinic	43	11.84
School	36	9.91
Physiotherapy Institute	32	8.81
Studio for small groups	30	8.26
Club Health and Fitness	28	7.71
Heart Rehabilitation Institute	11	3.03
Private medical center	11	3.03
Other	43	11.84
^†^Type of work		
Clinical Therapy	159	43.8
Physical Training	52	14.3
Management	48	13.2
Health promotion	35	9.6
Education/Physical Education	34	9.4
Research	7	1.9
Other	28	7.7
Role		
Physician	75	20.66
Physiotherapist	62	17.07
Nutritionist	53	14.6
Nurse	51	14.04
Teacher	48	13.22
Lecturer of training or Health Promotion	18	4.95
Physiologists	16	4.4
Other	61	16.8

SD, standard deviation; ^†^Total participants are larger than 363 because 139 of the participants work in more than one type of workplace/one type of profession. Accordingly, the cumulative percentage is over 100%.

### Scale’s psychometric properties

Cronbach’s alpha Index, indicating internal reliability, demonstrated the survey’s acceptability with a score of 0.75. Omitting variables did not indicate that any items negatively affected the scale’s internal consistency. The interrater test-retest reliability of the scale was excellent: ICC = 0.98 (95% confidence interval, 0.91-0.99).

Regarding factor structure analysis, the K1 criterion and scree plot resulted in a three-factor solution explaining 44.18% of the questionnaire’s variance. An initial examination of the items using PCA revealed no low communality (> 0.40) issues, thus no item was removed. For factor analysis results, refer to online supplement number 2.

### The importance of physical activity for the health of patients

Notably, all survey participants unanimously emphasized the crucial need to integrate physical activity into the medical care system for individuals with T2D, providing an unanimous rating of 5.0 on a scale ranging from 1.0 to 5.0 (**Table 2**).

**Table 2:** Attitudes of professionals regarding physical activity in people with diabetes (n = 363).

Variable	Median (percentile 25-75) or n (%)	chi-square (statistical significance)
The importance of physical activity for the health of patients: median (percentile 25-75)		
The collateral damage to public health due to sedentary behavior in the corona routine may be higher than the disease itself (1-disagree at all, 5- strongly agree)	4.0 (3.0-5.0)	--------------
Physical activity should be integrated into the medical care system for diabetics (1- disagree at all, 5- very agree)	5.0 (5.0-5.0)	--------------
Professional awareness and guidance for physical activity among diabetics: median (percentile 25-75)		
Diabetics are aware of the importance of physical activity as part of the treatment and prevention of the complications of the disease (1-not at all aware, 5- are very aware)	3.0 (2.0-3.0)	--------------
Physicians usually recommend daily physical activity for diabetics (0-not at all, 4-yes, regularly)	2.0 (2.0-3.0)	--------------
The role of the physician in prescribing physical activity recommendations to diabetics: n (%)		
yes, it's part of the physician's job yes, but the physician doesn't have enough time Yes, but the physician does not have the necessary knowledge No, it's not the physician's job	92.0 (25.3) 60.0 (16.5) 102.0 (28.1) 109.0 (30.0)	15.5 (0.0014)
The most qualified professional to prescribe physical activity for diabetics: n (%)		
Physician	57.0 (15.7)	122.5 (<0.0001)
Exercise physiologist	109.0 (30.0)	
Physiotherapist	103.0 (28.4)	
Physical Education Teachers	16.0 (4.4)	
Fitness and Health Coaches	52.0 (14.3)	
Other	26.0 (7.2)	
The most suitable framework for controlling PA programs for diabetics: n (%)		
Rehabilitation Institutes	42.0 (11.6)	102.6 (<0.0001)
Framework in the community	62.0 (17.1)	
health maintenance organization	120.0 (33.1)	
Independently-monitored and remote accompaniment	115.0 (31.7)	
Independently without the need for accompaniment	24.0 (6.6)	
National Infrastructures and Information: median (percentile 25-75)		
The explanation in the health system for the importance of physical activity for diabetics is effective (1-ineffective at all, 5- very effective)	3.0 (2.0-4.0)	--------------
The key ingredient to being reinforced in the program for diabetics: n (%)		
Infrastructure for tailored physical activity	100.0 (27.5)	252.76 (<0.0001)
Multi-professional consulting	208.0 (57.3)	
Remote monitoring and control system	51.0 (14.0)	
Other	4.0 (1.1)	
Who should fund physical activity for diabetics: n (%)		
Health maintenance organization as part of the healthcare basket	280.0 (77.1)	314.8 (<0.0001)
Insurance company	51.0 (14.0)	
Private funding of the patient	32.0 (8.8)	
The main factor preventing diabetics from exercising regularly: n (%)		
Lack of responsiveness	159.0 (43.8)	219.0 (<0.0001)
Lack of awareness of the importance of activity	121.0 (33.3)	
Lack of accessibility to activity centers	34.0 (9.4)	
Economic barriers	27.0 (7.4)	
Healthcare staff do not recommend	22.0 (6.1)	

*The significant value notes are set to 0.05.

### Professional awareness of guidance for PA for people with diabetics

**Figure 1:**
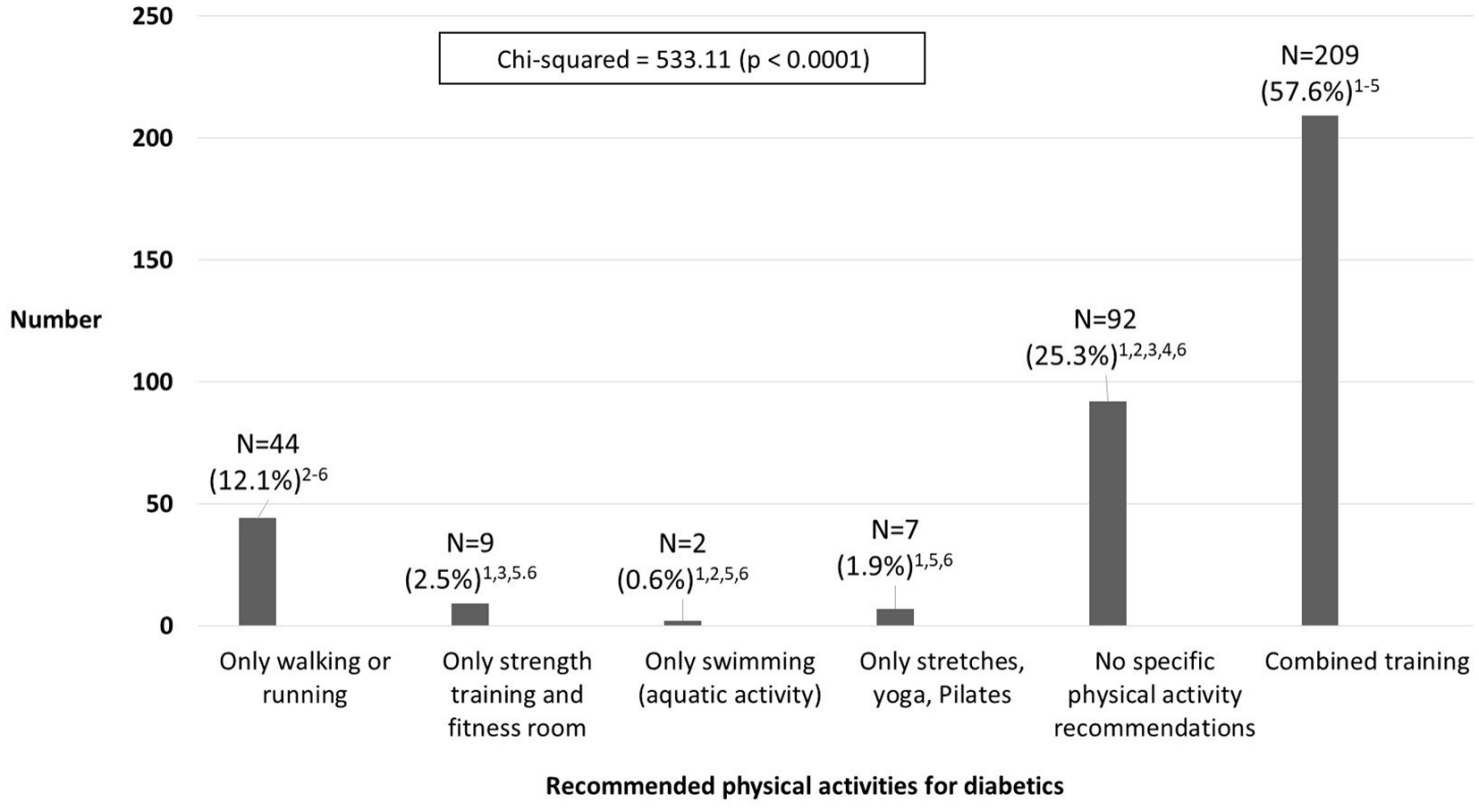
Recommended physical activities for diabetics. Statistical significantly different from "just walking or running"; (2) Statistically significantly different from "just strength training and gym activity"; (3) Statistical significant different from "Only Swimming (Activity in Water)"; (4) Statistically significantly different from "Just Stretching, Yoga, Pilates"; (5) Statistically significantly different from "No recommendations for a particular activity"; (6) Statistically significantly different from "integrated training”.

The survey revealed diverse opinions regarding the preferred exercises for individuals with diabetes. A majority (57.6%) advocated for a combination of exercises encompassing multiple physical fitness components. Conversely, a 25.3% emphasized the importance of general physical activity, deeming specific exercise recommendations unnecessary. A minority (12.1%) suggested a focus on walking or running as the primary training regimen (**Figure 1**).

### The most qualified professional to prescribe PA for diabetics

As reported in **Table 2**, exercise physiologists emerged as the most qualified group for making physical activity recommendations (30%), closely followed by physiotherapists (28%). In contrast, physicians (15.7%) and fitness and health coaches (14.3%) lagged behind, with other professions each comprising less than 10%. Notably, respondents highlighted the infrequency with which physicians recommend physical activity for diabetic patients.

### The most suitable framework for controlling the PA program for diabetics and national infrastructures and information

Within the survey context, opinions on the most suitable framework for managing PA programs for individuals with T2D varied. A notable 33.1% of respondents favored HMOs, closely followed by 31.7% endorsing independently-monitored and remote accompaniment. Additionally, 17.1% preferred a community-based framework, 11.6% supported rehabilitation institutes, and only 6.6% backed independent participation without the need for accompaniment (**Table 2**).

### The key ingredient to being reinforced in the program for diabetics and who should fund PA activity for diabetics

In relation to HMOs, approximately 77% of survey participants advocated for structuring funding for PA as part of the Medical Services basket within the Healthcare Government’s financial plan. The median score regarding the effectiveness of information in the health system about the importance of PA was 3.

Furthermore, a majority of respondents (57.3%) emphasized the importance of reinforcing PA recommendations as part of a multidisciplinary clinical team. Additionally, 27.5% highlighted the need to strengthen infrastructures tailored to individual PA needs and supervised training by professionals in the field (refer to **Table 2** for details).

### The main factor preventing diabetics from conducting PA regularly

Respondents’ opinions indicate that patients possess partial awareness of the importance of PA for individuals with T2D (median score of 3 out of 5). Furthermore, 43.8% of respondents identified the primary factor contributing to this lack of awareness as a lack of responsiveness, followed by a lack of awareness regarding the importance of physical activity (33.3%). Other factors included limited accessibility to activity centers (9.4%), economic barriers (7.4%), and 6.1% of respondents noted that healthcare staff do not recommend physical activity (**Table 2**).

### Professional roles and perceptions in physical activity guidance

**Table 3** presents differences in professional attitudes toward PA in the context of diabetes care (ordinal scale variables). Significant differences were found between health professions on several items. Health professions lecturers and physical education teachers/trainers expressed the highest agreement with the statement that sedentary behavior during the COVID-19 pandemic poses a greater secondary public health risk than the disease itself (Kruskal-Wallis test = 16.93 (p < 0.01). In addition, physicians and physical education teachers/trainers reported the highest levels of agreement with the statement that physicians routinely recommend PA to people with diabetes (Kruskal-Wallis test = 22.55, p<0.01). Interestingly, as reported in **Table 4**, in all health professions there was a high agreement that (64-88%) HMOs national health basket should fund PA for people with diabetes.

**Table 3:** Professional attitudes toward physical activity in people with diabetes – ordinal scale variables.

Question	^7^ Health Professions Lecturer (N=18): Median (25th to 75th percentile) [mean location]	^6^Physical Education Teacher/Trainer (N=48): Median (25th to 75th percentile) [mean location]	^5^Physiotherapist (N=62): Median (25th to 75th percentile) [mean location]	^4^Exercise physiologist (N=16): Median (25th to 75th percentile) [mean location]	^3^Dietitian (N=53): Median (25th to 75th percentile) [mean location]	^2^Nurse (N=51): Median (25th to 75th percentile) [mean location]	^1^Physician (N=75): Median (25th to 75th percentile) [mean location]	Kruskal-Wallis test
The secondary harm to public health due to sedentary behavior during the COVID-19 routine may be greater than the disease itself (1 – Strongly disagree, 5 – Strongly agree)	5 (4.25-5.00) ^1^[226.07]	5 (4.00-5.00) ^1,3,5^[217.77]	4 (3.50-5.00) ^6^[178.10]	4.5 (4.00-5.00) [198.12]	4 (3.00-5.00) ^6^[170.79]	4 (3.00-5.00) [178.64]	4 (3.00-5.00) ^6,7^ [150.03]	16.93*
Physical activity should be integrated into the medical care (1 – Strongly disagree, 5 – Strongly agree)	5 (5.00-5.00) [201.00]	5 (5.00-5.00) [184.59]	5 (5.00-5.00) [191.90]	5 (5.00-5.00) [167.75]	5 (5.00-5.00) [194.36]	5 (5.00-5.00) [162.33]	5 (5.00-5.00) [171.64]	4.57
People with diabetes are aware of the importance of physical activity as part of disease treatment (1 – Not aware at all, 5 – Very aware)	3 (2.25-3.00) [152.29]	3 (2.00-3.00) [169.85]	3 (2.00-3.00) [178.35]	2.5 (2.00-3.00) [134.92]	3 (2.00-3.00) [166.42]	3 (3.00-3.00) [199.53]	3 (3.00-4.00) [204.95]	9.92
Physicians routinely recommend daily physical activity to people with diabetes (0 – Not at all, 4 – Yes, regularly)	2 (1.25-2.00) ^1,6^ [103.50]	3 (2.00-3.00) ^3,4,5,7^[203.43]	2 (2.00-3.00) ^1,6^[164.36]	2 (1.00-2.00) ^1,6^ [113.71]	2 (1.75-3.00) ^1,6^[163.90]	2 (2.00-3.00) ^1^[170.85]	3 (2.00-3.00) ^2,3,4,5,7^[215.05]	22.55*
Health system education on the importance of physical activity is effective (1 – Not effective at all, 5 – Very effective)	2 (1.00-2.00) [80.71]	3 (2.00-4.00) [184.00]	3 (2.00-3.00) [169.32]	3 (2.00-3.00) [162.37]	3 (2.00-3.25) [165.59]	3 (3.00-4.00) [231.12]	3 (2.00-4.00) [180.27]	10

The other group was omitted from the analysis; *The significance level was set at 0.05 and adjusted to 0.01 using the Bonferroni method(0.05/5 = 0.01); ^1^Significantly different from Physician”; ^2^Significantly different from “Nurse”; ^3^Significantly different from “Dietitian”; ^4^Significantly different from “Exercise Physiologist”; ^5^Significantly different from “Physiotherapist”; ^6^Significantly different from “Physical Education Teacher/Trainer”; ^7^Significantly different from “Health Professions Lecturer”; ^8^Significantly different from “Other”.

**Table 4:** Professional attitudes toward physical activity in people with diabetes – categorical variables.

Reported questions	Answers options	^7^Health Professions Lecturer (N=18): N (%)	^6^Physical Education Teacher/Trainer (N=48): N (%)	^5^Physiotherapist (N=62): N (%)	^4^Exercise physiologist (N=16): N (%)	^3^Dietitian (N=53): N (%)	^2^Nurse (N=51): N (%)	^1^Physician (N=75): N (%)
Recommended physical activity for people with diabetes	Only aerobic	5.00 (27.80)^3,4^	4.00 (8.33)^2^	7.00 (11.30)^2^	0 (0)^2,7^	2.00 (3.80)^7^	17.00 (33.30)^4,5,6^	8.00 (10.70)
	Strength only	0 (0)	4.00 (8.33)	0 (0)	1.00 (6.20)	1.00 (1.90)	0 (0)	1.00 (1.30)
	Stretching, yoga, or Pilates only	1.00 (5.60)	2.00 (4.16)	0 (0)	0 (0)	1.00 (1.90)	0 (0)	3.00 (4.00)
	No specific recommendations	1.00 (5.60)^2,3,5,6^	8.00 (16.66)^3,7^	16.00 (25.80)^7^	1.00 (6.20)^2,3^	23.00 (43.40)^4,6,7^	16.00 (31.40)^4,7^	14.00 (18.70)
	Combined training	11.00 (61.10)^2,4^	30.00 (62.50)^2,4^	39.00 (62.90)^2,4^	14.00 (87.50)^1-3,5-7^	26.00 (49.10)^4^	18.00 (35.30)^1,4-7^	49.00 (65.30)^2,4^
Physician’s role to provide physical activity recommendations for people with diabetes	Physician’s role	2.00 (11.10)^1,2^	13.00 (27.08)^4^	13.00 (20.96)^4^	0 (0)^1,2,4,5^	5.00 (9.40)^2^	20.00 (39.20)^3,4,7^	29.00 (38.70)^4,7^
	Physician–no time	2.00 (11.10)	7.00 (14.58)	8.00 (12.90)	1.00 (6.25)	9.00 (17.00)	8.00 (15.70)	17.00 (22.70)
	Physician-lacks knowledge	10.00 (55.60)^1,2,4-6^	14.00 (29.16)^7^	17.00 (27.41)^7^	3.00 (18.75)^7^	21.00 (39.60)	11.00 (21.60)^7^	20.00 (26.70)^7^
	Not physician	4.00 (22.20)^4^	14.00 (29.16)^4^	24.00 (38.70)^4^	12.00 (75.00)^1-3,5-7^	18.00 (34.00)^4^	12.00 (23.50)^4^	9.00 (12.00)^4^
The most qualified professional to provide physical activity guidance for people with diabetes	Physicians	2.00 (11.10)	8.00 (16.66)	3.00 (4.83)	0(0) ^1,2^	5.00 (9.43)	12.00 (23.52)^4^	17.00 (22.66)^4^
	Exercise physiologists	8.00 (44.40)^1,2,4,5^	19.00 (39.58)^1,4,5.7^	7.00 (11.29)^3,4,6,7^	14.00 (87.5)^1-3,5-7^	21.00(39.62)^1,4,5^	12.00 (23.52)^7^	12.00 (16.00)^7^
	Physiotherapists	1.00 (5.60)^1,2,5^	2.00 (4.16)^1,2,5^	46.00 (74.19)^1-4, 6,7^	1.00 (6.20) ^1,2,5^	11.00 (20.75)^5^	15.00 (29.41)^4-7^	23.00 (30.66)
	PE teachers	1.00 (5.60)	8.00 (16.66)	1.00 (1.61)	1.00 (6.20)	1.00 (1.88)	0 (0)	4.00 (5.33)
	Fitness trainers	3.00 (16.70)	11.00 (22.91)^4^	3.00 (4.83)	0 (0)^6^	8.00 (15.09)	8.00 (15.68)	13.00 (17.33)
The most appropriate setting for monitoring physical activity among people with diabetes	Rehabilitation centers	3.00 (16.70)^4^	10.00 (20.83)^4^	4.00 (6.50)^4^	7.00 (43.70)^1-3,5-7^	5.00 (9.40)^4^	6.00 (11.80)^4^	2.00 (2.70)^4^
	Community	2.00 (11.10)	11.00 (22.91)	5.00 (8.10)	2.00 (12.50)	15.00 (28.30)	9.00 (17.60)	15.00 (20.00)
	HMOs	8.00 (44.40)^4^	15.00 (31.25)^4^	27.00 (43.50)	3.00 (18.80)^5;7^	14.00 (26.40)	18.00 (35.30)	19.00 (25.30)
	Independently – remote monitoring	3.00 (16.70)^1,5^	11.00 (22.91)	24.00 (38.70)^7^	4.00 (25.00)	18.00 (34.00)	14.00 (27.50)	28.00 (37.30)^7^
	Independently – without support	2.00 (11.10)	1.00 (2.08)	2.00 (3.20)	0 (0)	1.00 (1.90)	4.00 (7.80)	11.00 (14.70)

Questions		**^7^Health Professions Lecturer (N=18):** **N (%)**	**^6^Physical Education Teacher/Trainer (N=48):** **N (%)**	**^5^Physiotherapist (N=62):** **N (%)**	**^4^Exercise physiologist (N=16):** **N (%)**	**^3^Dietitian (N=53):** **N (%)**	**^2^Nurse (N=51):** **N (%)**	**^1^Physician (N=75):** **N (%)**
The key component that should be reinforced in programs for people with diabetes	Infrastructure for physical activity	4.00 (22.20)	16.00 (33.33)	26.00 (41.90)	5.00 (31.20)	9.00 (17.00)	9.00 (17.60)	21.00 (28.00)
	Multidisciplinary counseling	12.00 (66.70)^5,6^	22.00 (45.83)^2,7^	24.00 (38.70)^1-3,7^	9.00 (56.20)	31.00 (58.50)^5^	35.00 (68.60)^5,6^	46.00 (61.30)^5^
	Remote monitoring	2.00 (11.10)^3^	8.00 (16.66)	12.00 (19.40)	2.00 (12.50)	13.00 (24.50)^7^	6.00 (11.80)	6.00 (8.00)
Who should fund physical activity for people with diabetes?	HMOs - national health basket	16.00 (88.90)	38.00 (79.16)	50.00 (80.60)	14.00 (87.50)	43.00 (81.10)	41.00 (80.40)	48.00 (64.00)
	Health maintenance organizations – supplementary insurance	2.00 (11.10)	6.00 (12.50)	9.00 (14.50)	1.00 (6.20)	10.00 (18.90)	5.00 (9.80)	13.00 (17.30)
	Private funding by the patient	0 (0)	4.00 (8.33)	3.00 (4.80)	1.00 (6.20)	0 (0)	5.00 (9.80)	14.00 (18.70)
The main barrier preventing individuals with diabetes from engaging in regular physical activity	Lack of patient adherence	9.00 (50.00)	14.00 (29.16)	30.00 (48.40)	3.00 (18.80)	28.00 (52.80)	24.00 (47.10)	37.00 (49.30)
	Lack of access to activity centers	2.00 (11.10)	5.00 (10.41)	4.00 (6.50)	3.00 (18.80)	3.00 (5.70)	2.00 (3.90)	11.00 (14.70)
	Lack of awareness of physical activity importance	6.00 (33.30)	23.00 (47.91)	19.00 (30.60)	8.00 (50.00)	15.00 (28.30)	19.00 (37.30)	17.00 (22.70)
	Financial barriers	0 (0)	5.00 (10.41)	3.00 (4.80)	0 (0)	4.00 (7.50)	3.00 (5.90)	7.00 (9.30)
	Healthcare professionals do not provide recommendations	1.00 (5.60)	1.00 (2.08)	6.00 (9.70)	2.00 (12.50)	3.00 (5.70)	3.00 (5.90)	3.00 (4.00)

Column 1 includes the reported questions. The second column indicates the answer options to the questions of column 1The other group was omitted from the analysis; *The significance level was set at 0.05 and adjusted to 0.007 using the Bonferroni method(0.05/7 = 0.007); HMO, health maintenance organizations; PE, physical education;^1^ Significantly different from Physician”; ^2^ Significantly different from “Nurse”; ^3^ Significantly different from “Dietitian”; ^4^ Significantly different from “Exercise Physiologist”; ^5^ Significantly different from “Physiotherapist”; ^6^ Significantly different from “Physical Education Teacher/Trainer”; ^7^ Significantly different from “Health Professions Lecturer”; ^8^ Significantly different from “Other”.

In **Table 4** (categorical variables), significant differences emerged across professions in the first five questions. With respect to the type of PA recommended for people with diabetes, the majority (> 50%) of health professionals recommended combined training; however, this recommendation was provided by less than half of nurses (N=18, 35.3%) and dietitians (N=26, 49.1%). The highest prevalence of professionals recommending combined training was found among exercise physiologists (N=14, 87.5%).

Professionals, views varied on the physician’s role in recommending PA. This responsibility was most commonly endorsed by physicians (38.7%) and nurses (39.2%), but not at all by exercise physiologists. In contrast, when asked who is most qualified to provide PA guidance, the majority of exercise physiologists (87.5%) and physiotherapists (74.1%) identified their own professions, whereas only 22.6% of physicians did the same. Regarding optimal settings for PA monitoring, exercise physiologists were distinct, with 43.7% favoring rehabilitation centers.

Regression analyses identified key predictors of these healthcare professionals’ attitudes. Professional background as an exercise physiologist significantly predicted both a greater likelihood of recommending combined aerobic and resistance training for individuals with diabetes (β = 0.35, p = 0.03), and increased agreement with the view that PA counseling is not the physician’s primary role (β = 0.16, p = 0.046). Additionally, profession strongly influenced perceptions of qualification: exercise physiologists and physiotherapists were significantly more likely to view their own profession as most qualified to provide PA guidance (β = 0.51, p < 0.001; β = 0.28, p = 0.004, respectively).

## Discussion

This study aimed to explore the attitudes and knowledge of healthcare professionals concerning PA for patients with T2D. Understanding these attitudes is crucial, given the pivotal role healthcare professionals play in promoting PA, especially among populations at heightened risk of poor health due to physical inactivity, such as individuals with T2D.

Routine assessment of PA levels and brief practical interventions delivered by healthcare professionals such as providing advice or counseling can effectively influence patient behavior by encouraging the adoption and maintenance of healthy lifestyle habits (21). Moreover, understanding the perspectives and knowledge of experts on PA in the context of T2D offers valuable insights for shaping national strategies to promote PA (22). The following discussion explores how the attitudes of healthcare professionals, institutions, and policymakers intersect to support the integration of PA into care for individuals with T2D.

Healthcare professionals advocate for providing prescribed PA services for diabetes within the health system and the health basket (Medical Government’s financial plan). Accordingly, recent healthcare policies in several countries highlight the role and responsibility of health systems and governments in implementing measures targeting at-risk populations to prevent no communicable diseases (23, 24). However, guidelines specific to the position and professionals’ perceptions of PA in coping with and treating patients with T2D are yet to be published (25).

The findings of the present study align with international best practices emphasizing the central role of PA in the prevention and management of T2D. Leading global health organizations, including the World Health Organization (WHO) (5), the American Diabetes Association (ADA) (6), the European Association for the Study of Diabetes (EASD) (7), and the ACSM (26), advocate structured lifestyle interventions as a first-line strategy for addressing T2D.

Health professionals’ opinions are generally consistent with health organizations recommendations: for adults to engage in 150–300 minutes of moderate-intensity aerobic physical activity per week to reduce the risk of non-communicable diseases, including TD2 (5). Evidently, the ADA/EASD consensus report emphasizes PA as an essential component of comprehensive diabetes care, encouraging routine integration of exercise counseling and support into clinical practice (27). Moreover, the ADA’s position statement emphasizes that supervised and individualized exercise interventions, such as aerobic and resistance training, are more effective than non-supervised programs in improving adherence, cardiorespiratory fitness, and metabolic outcomes in individuals with diabetes (4). Evidence from recent meta-analyses demonstrates that supervised aerobic or resistance training significantly reduces glycated hemoglobin (A1C) in adults with T2D, even in the absence of dietary changes. In contrast, unsupervised exercise leads to meaningful A1C reductions only when paired with a dietary co-intervention (28).

Furthermore, individuals who engage in supervised aerobic and resistance training experience greater improvements across several health markers, including body mass index (BMI), waist circumference, blood pressure, cardiovascular fitness, muscular strength, and HDL cholesterol levels (29).

Consistent results and recommendations are observed in multi-participant studies conducted in various countries (30–32). These studies highlight the significant contribution of lifestyle changes, particularly moderate-intensity PA, in preventing the development of T2D. Lifestyle modifications, incorporating PA, were shown to be notably effective, sometimes surpassing the efficacy of medication (30, 31).

The Look AHEAD (Action for Health in Diabetes) trial further supports these conclusions, demonstrating that intensive lifestyle interventions, including supervised exercise, lead to significant improvements in weight loss, cardiorespiratory fitness, blood glucose control, blood pressure, and lipid profiles (33).

Diabetic individuals can engage in safe and independent exercise, but attention must be given to various risks associated with diabetes complications, such as peripheral neuropathy, autonomic neuropathy, retinopathy, kidney disease, and cardiovascular disease (21, 22). Additionally, precautions for preventing hypoglycemia and hyperglycemia should be considered.

Specific recommendations include consuming carbohydrates before exercising when glucose levels are below 100 mg/dl, depending on the activity’s duration and intensity. Guidelines also address avoiding intense activity in situations of hyperglycemia when blood glucose levels are between 250-350 mg/dl and over 350 mg/dl (6, 34). It is crucial to acknowledge the high variability in glycemic response following physical exertion in people taking insulin, necessitating personally tailored recommendations, including potential medication changes by physician (35, 36).

Monitoring diabetic patients accordingly highlights the critical role of structured and professionally guided exercise, particularly in clinical populations managing T2D, and therefore supports the rationale for supervised PA programs, ideally integrated into healthcare institutions such as HMOs, rehabilitation centers, and diabetes clinics. Supervision not only enhances clinical outcomes but also addresses safety concerns, especially among high-risk diabetic patients who may have comorbidities such as neuropathy or cardiovascular disease.

Despite the clear evidence reported in literature, our findings, highlight a significant gap in national infrastructure, particularly the absence of formal guidelines and standardized training for healthcare professionals in PA prescription. While physicians are widely regarded as central to promoting physical activity, it was reported that they are lacking the necessary tools, time, and specific knowledge to provide individualized exercise recommendations (30). This disconnect underscores the critical need to align local health policy with established global standards by implementing structured PA promotion strategies, integrating training into medical education, and ensuring systemic support for interdisciplinary care delivery. The alignment of our results with international recommendations strengthens the case for adopting global best practices at the national level, while also pointing to areas where policy reform and professional development are urgently required to optimize diabetes care and prevention efforts.

### Professional awareness, barriers and challenges for physical activity among diabetics

In our study, healthcare professionals (Physicians and paramedical personnel) widely recognize the importance of integrating PA into the treatment of T2D. Physicians were identified as key contributors to prescribing PA, with 70% of respondents affirming this role (**Table 2**). However, translating these recommendations into clinical practice remains a significant challenge due to several interrelated barriers. Primary among these are time constraints, which limit physicians’ ability to provide comprehensive PA counseling during routine consultations. This aligns with findings by O’Regan et al. (30), who identified uncertainty around exercise prescription and limited consultation time as primary obstacles faced by general practitioners.

A 2023 Delphi study in the UK (37), revealed that while there was consensus on the importance of exercise for diabetes management, the specifics regarding type, duration, and modality of exercise prescription varied among healthcare and fitness professionals. This inconsistency can lead to confusion among patients and highlights the need for standardized guidelines and standardized personal education too.

A further impediment is the lack of institutional infrastructure and support for PA promotion within many healthcare settings. Physicians report insufficient access to tools for assessing patients’ PA levels and identifying personal barriers to activity. Moreover, there are notable gaps in knowledge regarding core components of PA prescription, including frequency, intensity, duration, and type of exercise (38). While continuing professional education can help bridge this gap, participation remains low due to systemic issues such as the absence of structured referral pathways and weak interdisciplinary coordination (39).

In addition to healthcare professional knowledge gaps and organizational inefficiencies, financial and reimbursement issues also undermine the implementation of PA interventions. In many healthcare systems, inadequate reimbursement for PA counseling discourages healthcare providers from investing time and effort in formal PA prescription (14, 40). In the absence of supportive policies and financial incentives, PA promotion is often deprioritized in favor of competing clinical responsibilities.

Professional divides in attitudes toward physical activity (PA) promotion within healthcare. Despite most survey participants identifying exercise physiologists (30%) and physiotherapists (28%) as the most suitable professionals for prescribing physical activity, this delegation of responsibility is evidently not reflected in current clinical practice (37). Whereas physicians and nurses often consider PA counseling to be within the physician’s responsibility, exercise physiologists are more likely to oppose this view, emphasizing their specialized expertise as the appropriate source for PA guidance. This self-perception was mirrored in the choice of the most qualified professional to deliver PA guidance, where both exercise physiologists and physiotherapists showed strong professional identification. The preference of exercise physiologists for rehabilitation settings also underscores a distinct clinical orientation compared to other professions. Regression analyses confirmed that healthcare professional background strongly influences both attitudes and perceived responsibilities related to PA.

While healthcare professionals are well-positioned to deliver specialized guidance, stronger interdisciplinary collaboration is needed to ensure continuity of care and the successful integration of PA into diabetes management plans.

Collectively, our findings underscore the need for comprehensive strategies that address not only individual level barriers such as knowledge and time constraints, but also institutional limitations, including inadequate training systems, fragmented care models, and the lack of reimbursement mechanisms. Addressing these challenges is essential for operationalizing the clinical value of PA in T2D care and aligning real-world practice with international standards and evidence-based guidelines.

### National infrastructures and information

In the present study healthcare professionals recognize the necessity of establishing a national infrastructure for health promotion and advocacy for T2D. Most respondents emphasize the need to reinforce a system providing multidisciplinary advice to diabetics, with the funding for such a program ideally coming directly from HMOs in the healthcare basket (77.1%).

Survey experts highlight the significance of addressing patient-related barriers, with the lack of responsiveness and lack of awareness and knowledge identified as primary obstacles to PA among individuals with T2D. This aligns with recent reviews, which identify personal, motivational, social, mental, clinical, and self-efficacy factors as the main barriers to performing PA and training among diabetic patients (41).

A substantial portion of respondents (41.8%) indicates a lack of awareness and knowledge among healthcare professionals regarding the importance of PA for individuals with diabetes. Enhancing awareness and guidelines for PA across various training tracks for physicians and paramedical professionals is crucial. Establishing multi-professional teams is advocated to promote healthy and active lifestyles.

**Figure 2:**
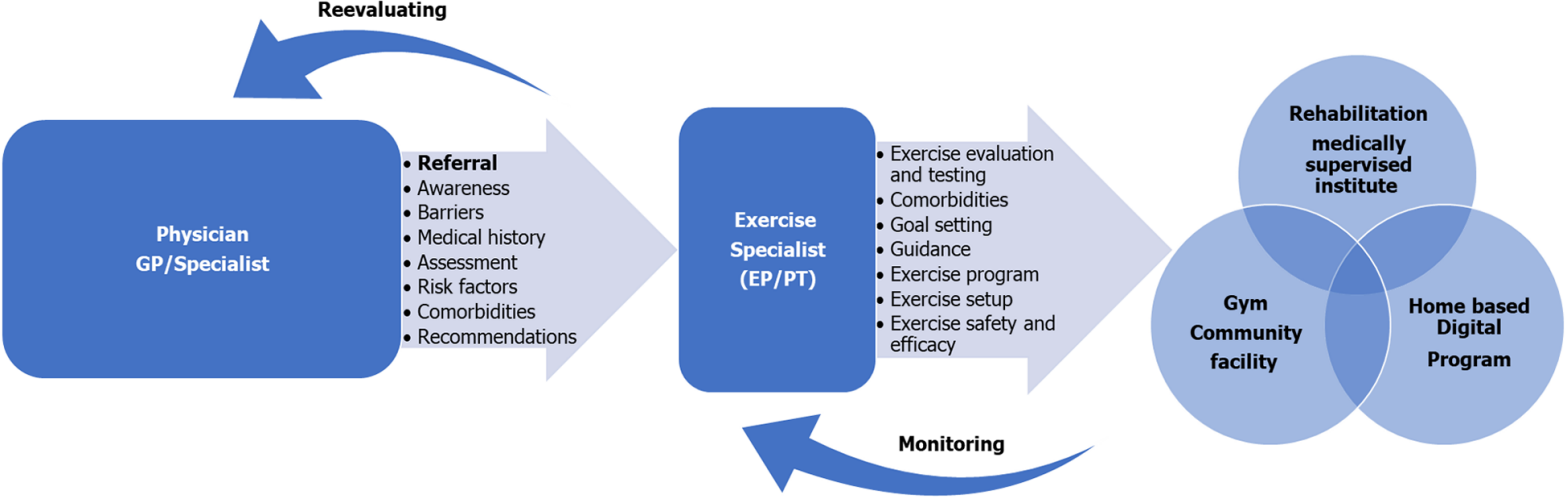
Physical activity as clinical practice care for patients with type 2 diabetics. Tailored Case Management Model incorporating PA for T2D Patients: Initiated by the attending physician [general practitioner (GP) or a specialist], referring the patient for an evaluation by an exercise physiologist (EP) or a physiotherapist (PT). This model features adaptable interventions tailored to each patient's specific needs. It aims for the formal recognition of Physical Activity (PA) as a national medical treatment program within the healthcare system, with the overarching goal of preventing and treating Type 2 Diabetes (T2D).

A comprehensive approach involving both doctors and nurses is pivotal in implementing and monitoring exercise adherence among individuals with diabetes while educating them about the benefits of exercise. This is particularly crucial when considering specific PA recommendations for individuals with T2D. To optimize patient care, we propose a tailored case management model with adaptable intervention approaches designed to meet each patient’s specific needs (**Figure 2**).

Our study population suggest that PA implementation could be included in HMOs services (33%). Additionally, PA could be conducted in medically supervised health centers, diabetes clinics, and independently at home, with remote monitoring and accompaniment (32%). Treatment funding in the healthcare basket (Medical Government’s financial plan) should be contingent on criteria such as the degree of risk, functional impairments, and the severity of the disease.

Our findings further support the need for a multidisciplinary approach to PA promotion, reflecting successful international models such as the Diabetes Prevention Program (DPP) (42), in the United States and the Finnish Diabetes Prevention Study (DPS) (31). In the DPP (42), a multi-professional response to the T2D PA program is exemplified. This program facilitates treatment, guidance, and follow-up in small groups, led by a team comprising certified medical trainers, exercise physiologists, clinical nutritionists, behavioral psychologists, and health educators. Similarly, the Finnish model DPS involves counseling sessions and nutrition and exercise guidelines (31), overseen by the attending physician, incorporating proactive sessions of circuit resistance training.These programs demonstrate the effectiveness of coordinated interventions delivered by interdisciplinary teams, including physicians, clinical exercise physiologists, dietitians, and behavioral specialists, in facilitating sustainable lifestyle changes among individuals at risk for or living with T2D.

Drawing from existing literature models and survey findings, there’s a clear need for personalized training programs tailored to patients with T2D (**Table 5**). Proposed diabetic intervention programs encompass three main tracks:

1. Initial Consultation for PA: tailored initial consultations for physical activity specific to individuals with T2D, conducted in HMOs initial clinics.
2. Home Monitoring Program and Remote Control: involve remote monitoring of physical activity, glycemic metrics, and patient adaptation to training. This program, administered in diabetes clinics, combines the expertise of exercise physiologists, clinical dietitians, and medical trainers (i.e., academically qualified exercise professionals with clinical credentials in training and managing chronic disease populations).
3. Structured and Supervised Rehabilitation Program: reserved to high-risk patients, this track entails a structured and supervised rehabilitation program in medical gyms. Overseen by exercise physiologists and/or physiotherapists, it provides a comprehensive approach to managing T2D through physical activity.

**Table 5:** Summary of recommendations for the implementation of exercise prescription, and glycemic balance in type two diabetics.

Type	Intensity (maximum predicted heart rate)	Duration (sessions per week/number of sets and repetitions for muscle group)	Frequency (Number of sessions per week)	Note
Daily activity; Daily steps cumulative measurement per day by activity clock, pedometer, mobile applications	according to personal ability, the Borg scale (1-3) medium-intensity 3000 steps or more. walking at a rate of 100 steps / per minute Borg scale (4-6)	Over 5000 steps per day, cumulatively average of at least 7500 steps per day. "Each minute counts"	Daily	Reducing prolonged and continuous sitting time over 2 hours. Breaking sitting times by walking for 2-10 minutes
Aerobics training; walking, running, riding, swimming, rowing, and elliptical	55-75% maximum heart rate or 40-60% heart rate reserve (Karvonen) Borg scale (4-6) Medium-intensity 75-90% maximum heart rate or 60-80% heart rate reserve (Karvonen) Borg scale (8-7)	At least 150-210 minutes per week ^*^Over 250 minutes for overweight and moderately obese patients or intensity between 90-125 minutes per week in high intensity level	3-5 sessions per week	Maintain continuous weekly persistence and no more than 2 consecutive days without physical training
Resistance training; strength/resistance exercises according to main muscle groups (legs, chest, back, shoulders, arms) Equipment: weights, straps, fitness elastic bands	Starting with 50-70% of 1 repetition maximum Borg scale (7-8) Advanced 70-85% of 1 repetition maximum Borg scale (7-8)	Starting 1-2 sets, 10-15 repetitions ^*^Rest time between sets 60-120 seconds	Starting 2 sessions per week Advanced: 2-3 sessions per week	Preference for choosing complex exercises for main muscle groups; Ensuring full control of exercises without muscle failure or overexertion.
Flexible training; stretching and mobility	Light to Moderate without pain	30-90 seconds per muscle group	2-3 sessions per week	Combined with aerobic or resistance training or not
Guidelines for implementation				
Target heart rate: The most common predictive formulas for maximum heart rate are: (220-age) or [207 – (0.67 X Age]. Borg scale 0-10 (rate of perceived exertion): Intensity of effort defined by subjective sensation in physical exertion is recommended in the range of 4-6 (slightly difficult to hard, around the anaerobic threshold value (7-8)).				

(4–7, 30, 34, 38).

Healthcare professionals in Israel unanimously agree on the crucial integration of physical activity (PA) into the treatment of type 2 diabetes (T2D) within the public healthcare system. Notably, 77% of survey participants supported allocating structured funding for PA by incorporating it into the national medical services basket (**Table 2**).

On the basis of literature data and ours too, a multidisciplinary approach is emphasized to disseminate and implement PA recommendations effectively, making them an integral part of patient care within clinics too. Collaboration between physicians and specialized teams, including exercise physiologists, trainers, and physiotherapists, is deemed essential for the successful promotion and incorporation of PA into the patient care system.

Considering the unique physiological, behavioral, and therapeutic requirements the establishment of a tailored case management model is strongly recommended. Initiated upon the attending physician’s recommendation, this model involves adaptable intervention approaches tailored to each patient’s specific needs. Formal recognition of PA as a national medical treatment program within the healthcare system is essential to realize these models, with the overarching goal of preventing and treating T2D patients.

### Study limitations

While this study provides a broad overview of PA integration into clinical care, it is based on self-reported data, which may be subject to bias or social desirability effects. The professional categories are not evenly represented which may affect potential non-response bias. Furthermore, the study does not evaluate how PA for individuals with T2D is prioritized in comparison to its use in the management of other chronic conditions. The use of convenience sampling may also limit the generalizability of the findings. Additionally, the survey does not offer a comparative analysis of exercise therapy versus other treatment modalities for diabetes. These limitations highlight the need for further investigation. Future research should include randomized, longitudinal studies to better assess implementation practices and outcomes across various clinical contexts.

## Conclusion

This study highlights the need for the National Healthcare System to strengthen its infrastructure for personalized PA programs targeting individuals with T2D. Our findings emphasize the importance of establishing multidisciplinary healthcare teams and allocating dedicated resources to implement and monitor tailored PA interventions, which are crucial for improving health outcomes in this population.

## Data Availability

The raw data supporting the conclusions of this article will be made available by the authors, without undue reservation.
