# Supplementary material for: Physical activity as clinical practice care for patients with type 2 diabetics and its implementation in routine clinical care: an expert opinion survey
Source: Front Endocrinol (Lausanne). 2025 Sep 11;16:1518285. doi: 10.3389/fendo.2025.1518285 (PMC12460137; doi:10.3389/fendo.2025.1518285)
Supplement: Supplementary file 1 [file DataSheet1.docx]

**Supplementary 1: Study Questioner Translation**

**National Policy for Integrating Physical Activity and Exercise as Part of the Treatment Regimen for Type 2 Diabetes**

Hello,

The following questionnaire is designed to help understand your attitudes regarding physical activity and exercise among Type 2 diabetes patients, with the aim of shaping a national policy for integrating physical activity and exercise as part of the treatment for the disease.

Your participation will assist in understanding the current situation in the country and in promoting the field for the development of comprehensive intervention approaches in the community.

Completing the questionnaire will take about 5 minutes (a total of 18 questions).

The questionnaire is anonymous, and your personal details will remain confidential.

We thank you for taking the time to participate in this survey.

----------------------------

**Indicate your age:**

Gender (circle one):

Woman

Man

**Relevant workplace (you can mark more than one answer):**

Hospital

Health Maintenance Organization (HMO)

Cardiac Rehabilitation Institute

Physiotherapy Institute

Private Clinic

Private Medical Center

Health and Fitness Club

Small Group Studio

Self-employed

School

Other

**Indicate the nature of your role:**

Management

Research

Health Promotion

Clinical Treatment

Education/Physical Education

Physical Training

Other

**Indicate years of experience in your profession:**

**Indicate your main profession:**

Doctor

Nurse

Dietitian

Physiologist

Physiotherapist

Physical Education Teacher

Lecturer in Health and Training Professions

Other

**To what extent do you agree with the following statement:** The secondary damage to public health, due to sedentary behavior and lack of physical activity, in the Corona crises may be higher than the disease itself (1-Strongly disagree, 5-Strongly agree):

1 Strongly disagree

2

3

4

5 Strongly agree

**In your opinion, on a scale of 1-5, to what extent should physical activity be integrated into the medical treatment plan for diabetes patients** (1-Strongly disagree, 5-Strongly agree):

1 Strongly disagree

2

3

4

5 Strongly agree

**In your work, what physical activity is advisable to recommend for diabetes patients** (you can mark more than one answer):

Walking or running

Strength training and gym activities

Swimming and water activities

Moderate exercise, stretching, yoga, Pilates

I won't recommend a specific physical activity, the main thing is to do any activity

**Indicate on a scale of 1 to 5 how aware you think diabetes patients in Israel are of the importance of physical activity as part of the treatment and prevention of disease complications and secondary damage to target organs** (1 - Not at all aware, 5 - Very aware):

1 Not at all aware

2

3

4

5 Very aware

**In your opinion, do doctors tend to recommend daily physical activity to diabetes patients?**

Yes, regularly

Yes, often

Yes, sometimes

Yes, rarely

Not at all

**State your opinion, is it the treating physician's role to give detailed recommendations on how to perform physical activity for diabetes patients?**

Yes, it's part of the doctor's role

Yes, but the doctor doesn't have enough time for it

Yes, but the doctor doesn't have the necessary knowledge for it

No, it's not the doctor's role to give instructions on how to perform physical activity

**In your opinion, which of the following professionals is most qualified to give instructions (prescriptions) for physical activity and training for diabetics?**

- Doctors
- Exercise physiologists
- Physiotherapists
- Physical education teachers
- Fitness and health trainers (gym)
- Other

**In your opinion, what is the most appropriate framework for monitoring and following up on physical activity and training for diabetics?**

- In rehabilitation institutes, such as cardiac rehabilitation
- In settings outside the medical system in the community, such as authorized gyms and community centers
- Within health maintenance organization (HMO) services
- Independently, at home or outside, with remote monitoring and guidance
- Independently, at home or outside, without the need for special guidance

**In the current state of the healthcare system, what is the main component (variable) that you think should be strengthened in an intervention program for diabetics?**

- Infrastructure for adapted and controlled physical activity
- Multi-professional counseling: physiological / nutritional / clinical / nursing / rehabilitative
- Remote monitoring and control systems
- Other

**In your opinion, who should finance physical activity for diabetics?**

- HMO within the health basket
- HMO within the framework of supplementary insurance
- Private funding by the patient

**In your opinion, what is the main factor preventing diabetics from engaging in regular physical activity?**

- Lack of compliance and internal motivation of the patient
- Lack of accessibility to dedicated activity centers for diabetics
- Lack of awareness and education about the importance of physical activity
- Economic barriers to enrolling in various paid activity frameworks
- Doctors and healthcare staff do not recommend physical activity enough

**In your opinion, on a scale of 1-5, rate how effective the education in the healthcare system is regarding the importance of physical activity for diabetics** (1- not effective at all, 5- very effective)

1 Not effective at all

2

3

4

5 Very effective

**Supplementary 2: Supplemental digital tent 2 - Factor structure of the scale developed - exploratory factor analysis**

| **Question number** | **Items** | **Factor Loadings** | | | **Cumulative variance explained** |
| --- | --- | --- | --- | --- | --- |
|  |  | **Factor 1**  Importance of Physical activity and activity type | **Factor 2**  Qualified personal and setting | **Factor 3**  Components need to be reinforced |  |
| 1 | The secondary health impact of sedentary behavior during the COVID-19 pandemic may be greater than the direct effects of the virus itsel | 0.82 | - | - | - |
| 3 | Recommended physical activty | 0.80 | - | - | - |
| 4 | Individuals with diabetes level of awareness regarding the importance of physical activity for managing their condition | 0.81 | - | - | - |
| 5 | Do physicians routinely recommend physical activity to patients with diabetes? | 0.78 | - | - | - |
| **Variance explained** | | **23.76%** | - | - | **23.76%** |
| 2 | The extent to which physical activity should be integrated into the treatment protocol for patients with diabetes | - | 0.41 | - | - |
| 6 | Physician’s role to provide specific instructions for physical activity | - | 0.55 | - | - |
| 7 | Profession most qualified to provide physical activity guidance to patients with diabetes | - | 0.80 | - | - |
| 8 | The most appropriate setting for monitoring physical activity | - | 0.52 | - | - |
| **Variance explained** | | - | **11.21%** | - | **34.97%** |
| 9 | What is the main component of the healthcare system that should be strengthened to support physical activity among patients with diabetes? | - | - | 0.65 | - |
| 10 | Who should be responsible for funding physical activity for patients with diabetes? | - | - | 0.67 | - |
| 11 | What is the main factor preventing patients with diabetes from engaging in regular physical activity? | - | - | 0.61 | - |
| 12 | How effective is the health system's education in promoting the importance of physical activity for patients with diabetes? | - | - | 0.59 | - |
| **Variance explained** | | - | - | **9.21%** | **44.18%** |
